# Transscleral cyclophotocoagulation followed by cataract surgery: a novel protocol to treat refractory acute primary angle closure

**DOI:** 10.1186/s12886-020-01483-0

**Published:** 2020-05-29

**Authors:** Wei Liu, Luning Qin, Chenjia Xu, Dandan Huang, Ruru Guo, Jian Ji, Nomdo M. Jansonius

**Affiliations:** 1 Tianjin Key Laboratory of Retinal Functions and Diseases, Tianjin International Joint Research and Development Centre of Ophthalmology and Vision Science, Eye Institute and School of Optometry, Tianjin Medical University Eye Hospital, 251 Fukang Road, Nankai District, Tianjin, 300384 China; 2 Department of Ophthalmology, University of Groningen, University Medical Center Groningen, Groningen, The Netherlands

**Keywords:** Acute primary angle closure, Transscleral cyclophotocoagulation, Ultrasound biomicroscopy

## Abstract

**Background:**

To introduce a novel protocol to treat refractory acute primary angle closure (APAC): transscleral cyclophotocoagulation (TCP) followed by cataract surgery.

**Methods:**

Thirteen APAC eyes (13 patients) were enrolled in this prospective case series as study group. All patients underwent emergency TCP (20 pulses of 2000 mW during 2000 ms applied to the inferior quadrant) followed by scheduled cataract surgery. They were compared to 13 age- and gender-matched patients treated with emergency phacotrabeculectomy. We recorded intraocular pressure (IOP), best corrected visual acuity (BCVA), and complications, and several ultrasound biomicroscopy (UBM) parameters before and after TCP.

**Results:**

In the study group, IOP decreased from 51.5 ± 7.0 mmHg (mean ± standard deviation) before TCP to 16.4 ± 5.4 mmHg 1 day after TCP (*P* < 0.001). At 6 months, there was no significant difference in IOP between the study group (14.0 ± 3.4 mmHg) and control group (16.7 ± 4.3 mmHg; *P* = 0.090); IOP lowering medications were used by 0/13 in the study group and 2/13 patients in the control group (*P* = 0.48). At 6 months, there was no significant difference in BCVA between the study group and the control group (20/25 (20/200 to 20/25) and 20/30 (20/50 to 20/25), respectively; *P* = 1.0). The UBM parameters anterior chamber depth (*P* = 0.016), angle-opening distance at 500 μm (*P* = 0.011), and maximum ciliary body thickness (*P* < 0.001) increased significantly while the iris-ciliary process distance decreased significantly (*P* = 0.020) after TCP.

**Conclusions:**

TCP effectively lowers IOP and modifies the anterior chamber morphology in APAC; TCP followed by cataract surgery can be considered an alternative to treat refractory APAC but needs further evaluation.

**Trial registration:**

This project was registered in Chinese Clinical Trial Registry (ChiCTR1800017475) at July, 31, 2018 (http://www.chictr.org.cn/edit.aspx?pid=29629&htm=4).

## Background

Acute primary angle closure (APAC) is a common ophthalmic emergency with the rising rapidly to extremely high levels because of a sudden obstruction of the anterior chamber angle [1]. APAC patients often have typical symptoms, including blurred vision, ocular pain, headache, nausea, and vomiting. Immediate aggressive management is required to prevent irreversible damage to the eye and optic nerve and to relieve the excruciating symptoms of the patient [2]. However, the current treatment options for APAC do not always timely result in a sufficiently lowered IOP.

The current treatment strategy for APAC is to lower IOP by both topical and systemic medications followed by laser peripheral iridoplasty or iridotomy, and finally removal of the lens. However, this strategy has several limitations. The IOP of a significant proportion of APAC eyes cannot be controlled successfully by medical treatment, while laser application is often limited by corneal edema [3]. If the conventional treatment fails to reduce the IOP, surgical procedures in the acute phase have to be considered. However, performing acute surgery on APAC eyes is technically challenging and may increase the risk of complications because of the presence of corneal edema, inflammation, shallow anterior chamber, floppy iris and unstable lens [3].

In our previous study [4], and some other reports [5–7], diode laser transscleral cyclophotocoagulation (TCP) showed its IOP-lowering effects in eyes with APAC. However, the underlying mechanism remains unclear. In this study, we aimed to evaluate the safety and efficacy of TCP followed by cataract surgery on treating APAC. We also compared several ultrasound biomicroscopy (UBM) parameters to explore the underlying mechanisms.

## Methods

### Patients

This project was registered in Chinese Clinical Trial Registry (ChiCTR1800017475). Consecutive, medically unresponsive APAC patients (study group) from Tianjin Medical University Eye Hospital were prospectively enrolled. All the patients underwent emergency TCP followed by cataract surgery and were followed for 6 months. They underwent a thorough ocular examination, including an IOP measurement, a measurement of the best-corrected visual acuity (BCVA), slit-lamp biomicroscopy, ultrasonography B-scan, and UBM, before and 1 day after TCP. Any complications during and after the TCP were noted. IOP was measured by non-contact tonometry (Full Auto Tonometer TX-F, Canon, Japan) three times and the average value was recorded. This study was approved by the ethics committee of Tianjin Medical University Eye Hospital (2018KY-03) and followed the tenets of the Declaration of Helsinki. Informed consent was obtained from all subjects after explanation of the nature and possible consequences of the study.

Thirteen age- and gender-matched medically unresponsive APAC patients (control group) who underwent emergency phacotrabeculectomy (the historical default approach in our hospital) without TCP were retrospectively collected. The clinical outcome was compared between the two groups.

### Transscleral cyclophotocoagulation

All TCPs were performed by the same experienced surgeon (WL). TCP was performed using the OcuLight SLx 810 nm diode laser photocoagulator with the handheld fiberoptic G-probe (Iris Medical Instruments, Mountain View, CA, USA) under local anesthesia (2 ml of 2% lidocaine as a retrobulbar injection). The same laser protocol was used for all the patients: power 2000 mW, duration 2000 ms, number of applications 20. Laser applications were spaced evenly over the inferior quadrant (90 degrees), which implies overlapping rather than strictly adjacent shots. This deviates from the default approach in non-acute, open-angle situations, where the laser spots are given over 360 degrees – an approach that did not lower the IOP sufficiently in medically unresponsive APAC eyes in an earlier study [4]. We hypothesized that shrinkage of the ciliary body could be the mechanism and if so, a high dose applied to only a part of the ciliary body should be sufficient whilst having the advantage that the total dose would not increase, thus avoiding excessive inflammation. We chose the inferior quadrant for this, as that quadrant is the deepest in a normal eye, being the logical place to treat in case of closure. After TCP, patients got tobramycin and dexamethasone eye drops (TobraDex, S.A. Alcon-Couvreur N.V., Rijksweg, Puurs, Belgium) 4 times per day during the interval between TCP and the following cataract surgery.

### Ultrasound biomicroscopy and analysis

The anterior chamber configuration was determined before and 1 day after TCP using UBM (Suoer SW-3200 L; Tianjin, China) and a 50-MHz transducer probe by one experienced operator (LQ). Examinations were performed under the same room illumination and using the same fixation target on the ceiling for the fellow eye, with the patient in the supine position. After topical anesthesia, a plastic eyecup filled with physiologic saline was mounted on the globe without compressing the globe. Images were obtained at the center of the pupil, at the superior and inferior angles of the anterior chamber in the vertical meridian, and at the temporal and nasal angles of the anterior chamber in the horizontal meridian.

**Fig. 1:**
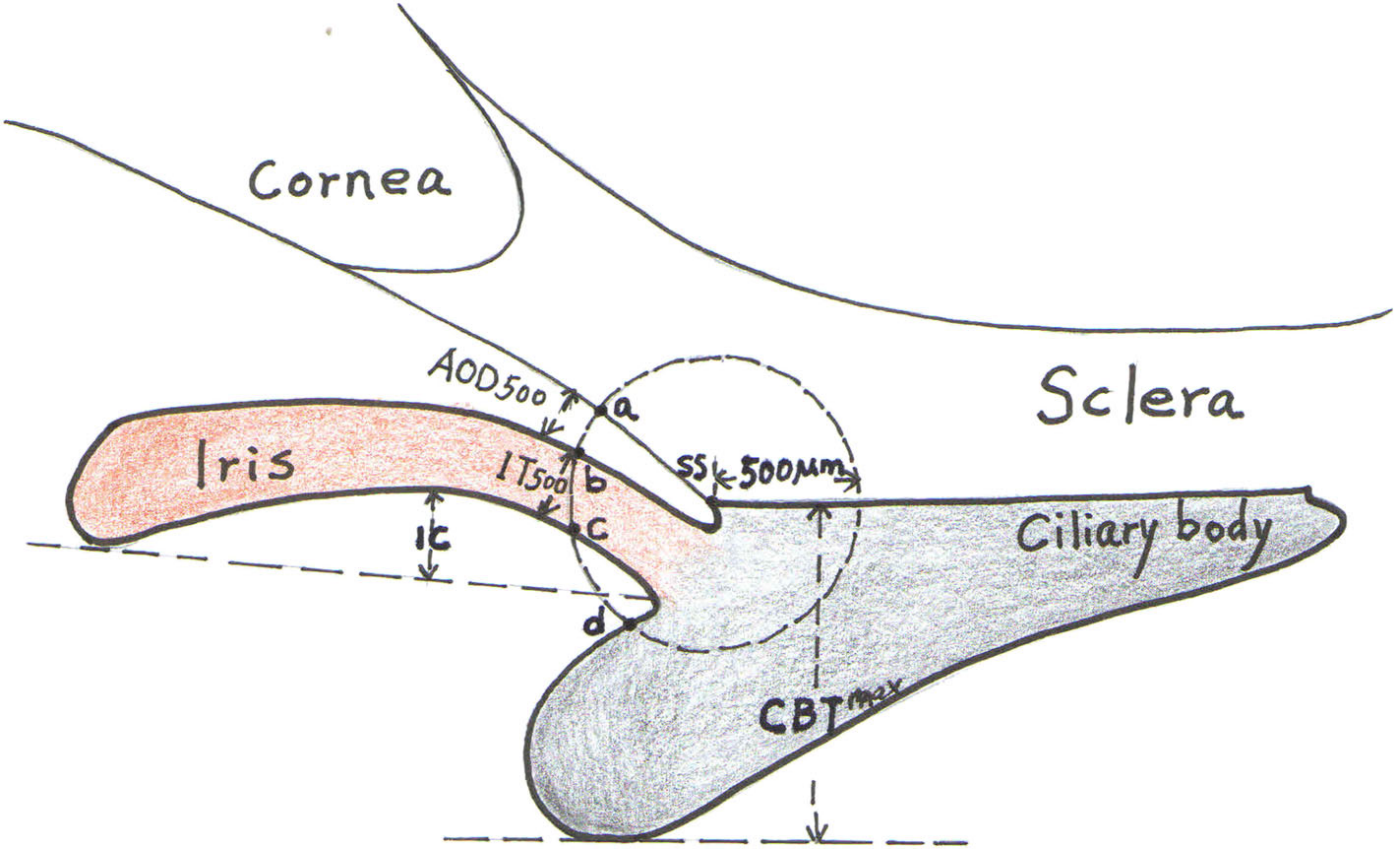
Schematic diagram of the ultrasound biomicroscopy parameters. A circle with a radius of 500 μm centered on the sclera spur (SS) is drawn. Angle-opening distance at 500 μm (AOD500) is the distance from corneal endothelium to the anterior iris perpendicular to a line drawn along the trabecular meshwork 500 μm from the SS. Iris thickness at 500 μm (IT500) is iris thickness at 500 μm from the SS (point b). Trabecular-ciliary process distance (TCPD) is a line extending from the corneal endothelium 500 μm anterior to the sclera spur (point a) toward the ciliary process (point d). Iris-ciliary process distance (ICPD) is the posterior surface of the iris 500 μm anterior to the sclera spur (point c) toward the ciliary process (point d). Maximum ciliary body thickness (CBTmax) is the distance from the most inner point of the ciliary body to the inner wall of sclera or its extended line. Iris curvature (IC) is the perpendicular distance from a line between the most central to the most peripheral points of the iris pigment epithelium to the posterior iris surface at the point of greatest convexity

All captured UBM images were analyzed using the software by a single observer (WL), who was masked to all clinical data, including whether a particular measurement was performed pre- or post-TCP. The UBM parameters measured in this study included the traditional parameters defined by Pavlin et al. [8, 9] and newly developed parameters described in recent studies [10–15]. From the images centered on the pupil, two parameters were determined: (1) anterior chamber depth (ACD; the axial distance from the corneal endothelium to the anterior lens surface) and (2) pupil diameter (PD; the shortest distance between the pupil edges of the iris cross-sections). From the radial images at the superior, inferior, nasal, and temporal positions, six parameters were measured (Fig. 1): (1) angle-opening distance at 500 μm (AOD500; the distance from the corneal endothelium to the anterior iris perpendicular to a line drawn along the trabecular meshwork 500 μm from the sclera spur), (2) iris thickness at 500 μm (IT500; iris thickness at 500 μm from the sclera spur [point b]), (3) trabecular-ciliary process distance (TCPD; the length of a line extending from the corneal endothelium 500 μm anterior to the sclera spur [point a] toward the ciliary process [point d]), (4) iris-ciliary process distance (ICPD; from the posterior surface of the iris 500 μm anterior to the sclera spur [point c] toward the ciliary process [point d]), (5) maximum ciliary body thickness (CBTmax; the distance from the innermost point of the ciliary body to the inner wall of the sclera or its extended line), and (6) the iris curvature (IC; the perpendicular distance from a line between the most central to the most peripheral points of the iris pigment epithelium to the posterior iris surface at the point of greatest convexity).

### Cataract surgery

Cataract surgery was scheduled after TCP to definitely widen the narrow angle. Cataract extraction was performed after the resolution of cornea edema and anterior chamber inflammation. All the surgeries were performed by the same experienced surgeon (JJ). After topical anesthesia with 0.4% Oxybuprocaine Hydrochloride (Santen, Japan), a small limbal paracentesis was performed at the 2 o’clock and then a superior clear corneal incision was made with 3.0-mm keratome. The chamber was immediately deepened with ocular viscoelastic devices. After the continuous curvilinear capsulorhexis, the nucleus was removed with a standard four-quadrant, “divide and conquer” phacoemulsification technique. The cortical remnants was then removed, and the intraocular lens was placed in the capsular bag. The viscoelastic agent was completely aspirated from the anterior chamber at the end of the surgery.

### Phacotrabeculectomy

All the phacotrabeculectomies were performed by the same experienced surgeon (JJ). A two-site procedure was used. After peribulbar anesthesia with 2 ml of 2% lidocaine (Shandong Hualu, China), a fornix-based conjunctival flap and a rectangular scleral flap (3 by 4 mm in size) were performed. Mitomycin-C (Zhejiang Hanhui, China) 0.04% was applied for 3 min under the conjunctival and scleral flaps. Then, cataract surgery was performed as described above followed by trabeculectomy and peripheral iridectomy. The scleral flap was closed with two 10–0 nylon sutures (one of which was a releasable suture) and the conjunctival wound was closed with three 10–0 nylon sutures.

### Statistical analysis

Continuous variables were expressed as mean ± standard deviation (SD) after confirming normality of the data distribution (Shapiro-Wilk test, *P* > 0.05); if not normally distributed, we used median and interquartile range (IQR). The number of anti-glaucoma medications and the number of patients with BCVA better than 20/40 after surgery were compared between the two groups using Fisher exact test. Age, axial length, and IOP before and after surgery were compared using independent samples t test between the two groups. Pre- and post-TCP observations were compared using a paired samples t test or a Wilcoxon signed-rank test, for parametric and nonparametric data, respectively. A *P* value of 0.05 or less was considered statistically significant.

## Results

Thirteen APAC eyes of 13 consecutive patients refractory to medical treatment (including 1% pilocarpine, 2% carteolol, 0.2% brimonidine, 1% brinzolamide, oral methazolamide and intravenous 20% mannitol, if there were no contraindications) were prospectively enrolled in this study. All patients presented with typical APAC symptoms and signs, including blurred vision, severe ocular pain, headache, nausea, vomiting, corneal edema, unresponsive dilated pupil, shallow anterior chamber, and elevated IOP. All these patients underwent emergency TCP followed by scheduled cataract surgery (interval time: 5–10 days, median: 7 days). Table 1 presents the characteristics of the study group and control group.

**Table 1:** Baseline demographics of the study participants

	Study group	Control group	*P* value
Age (years)	67.6 ± 4.7 (58–75)	69.3 ± 3.9 (61–74)	0.16
Gender, female	9 (69%)	10 (77%)	0.50
Axial length (mm)	22.13 ± 0.61 (20.90–22.90)	22.25 ± 0.48 (21.13–22.87)	0.29
Eye, right	7 (54%)	6 (46%)	0.50

Presented as mean ± standard deviation (range) or number (percentage)

**Fig. 2:**
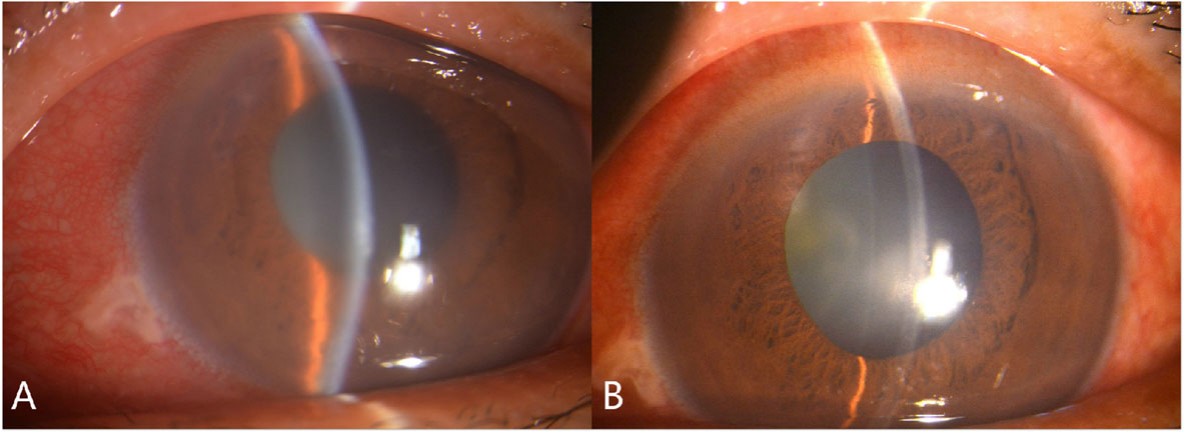
Anterior segment photograph before and after transscleral cyclophotocoagulation (TCP). A: severe conjunctival congestion and cornea edema before TCP. B: mild conjunctival congestion and clear cornea after TCP

In the study group, the IOP decreased significantly from 51.5 ± 7.0 mmHg (mean ± standard deviation) before TCP to 16.4 ± 5.4 mmHg at day 1 after TCP (*P* < 0.001). At day 1 after the TCP, the excruciating symptoms had disappeared and the corneal edema had been resolved in all patients (Fig. 2). The IOP of the study group before cataract surgery (17.2 ± 4.8 mmHg) was significantly lower than that of the control group before emergency phacotrabeculectomy (40.1 ± 5.7 mmHg; P < 0.001). At 6 months after surgery, the IOP was 14.0 ± 3.4 mmHg and 16.7 ± 4.3 mmHg in the study group and control group, respectively (*P* = 0.090). At 6 months after surgery, there were no patients in the study group and 2 patients in the control group who needed anti-glaucoma medications to control the IOP (*P* = 0.48).

In the study group, the BCVA of the patients ranged from hand movement to 20/50 (with a median value of finger counting) before TCP and improved in all patients at day 1 after TCP (ranging from finger counting to 20/30; median value 20/60). The single patient with a BCVA of finger counting after TCP was evidenced to have severe glaucomatous neuropathy when the cornea was clear enough to check the fundus. At 6 months after surgery, BCVA ranged from 20/200 to 20/25 (median value 20/25) in the study group and from 20/50 to 20/25 (median value 20/30) in the control group. There were 10 patients in the study group and 9 patients in the control group whose BCVA was better than 20/40 at 6 months after surgery (*P* = 1.0).

**Fig. 3:**
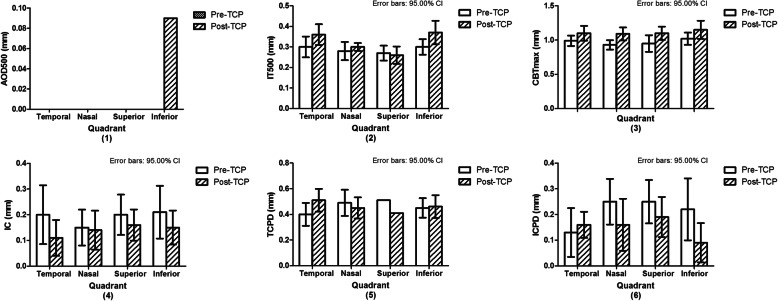
Comparison of UBM parameters in quadrants before and after transscleral cyclophotocoagulation (TCP). Error bars: 95% confidential interval. Error bars were not given for the nonparametric data (AOD500). See Fig. 1 for definitions of the parameters (AOD500, IT500, CBTmax, IC, TCPD, and ICPD)

**Fig. 4:**
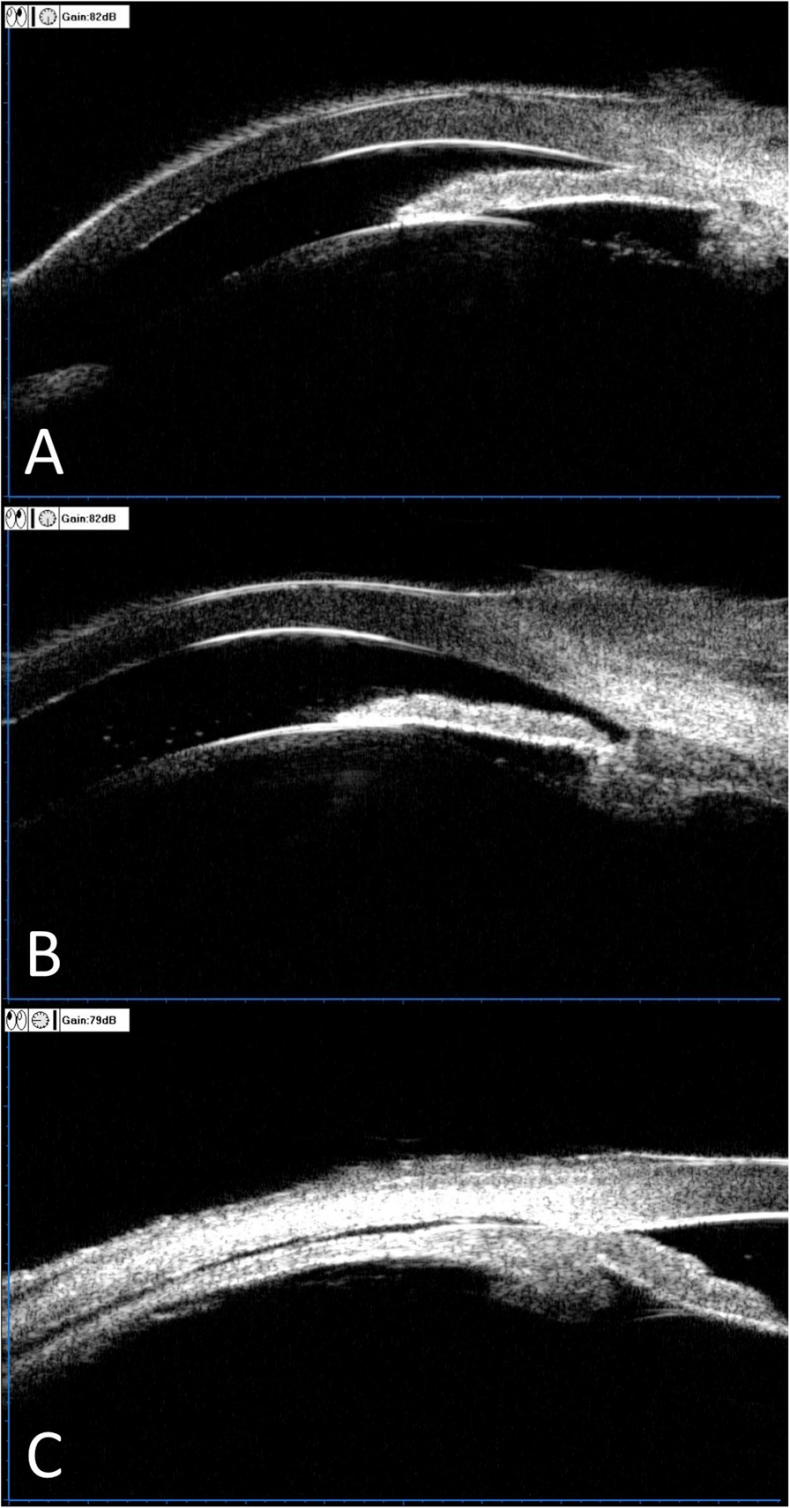
Ultrasound biomicroscopy image before and after transscleral cyclophotocoagulation (TCP). A: Shallow anterior chamber and closed inferior angle before TCP. B: Deepened anterior chamber and re-opened inferior angle after TCP. C: Ciliochoroidal detachment after TCP

Table 2 shows the effect of TCP on the 8 UBM parameters studied. A significant increase was found for ACD, AOD500, and CBTmax; a significant decrease was found for ICPD. Figure 3 shows the effect per quadrant for the 6 parameters that were studied for each quadrant separately. As can be seen in this figure, the global changes (as depicted in Table 2) in especially AOD500 and to a lesser extent also in ICPD were mainly due to changes in the, treated, inferior quadrant (Fig. 4) whereas the global change in CBTmax was due to changes in all quadrants.

**Table 2:** Comparison of UBM parameters before and after TCP

	Pre-TCP	Post-TCP	*P* value
ACD (mm)	1.62 ± 0.29	1.72 ± 0.30	0.016
PD (mm)	3.86 ± 1.32	4.10 ± 1.19	0.202
AOD500 (mm)	0.00 (0.00–0.00)	0.04 (0.01–0.08)	0.011
IT500 (mm)	0.29 ± 0.05	0.32 ± 0.04	0.095
CBTmax (mm)	0.98 ± 0.12	1.12 ± 0.15	0.000
IC (mm)	0.20 ± 0.15	0.14 ± 0.09	0.062
TCPD (mm)	0.46 ± 0.10	0.46 ± 0.05	0.960
ICPD (mm)	0.21 ± 0.10	0.15 ± 0.08	0.020

Presented as mean ± standard deviation or median (interquartile range); ACD: anterior chamber depth; PD: pupil diameter; AOD500: angle-opening distance at 500 μm; IT500: iris thickness at 500 μm; CBTmax: maximum ciliary body thickness; IC: iris curvature; TCPD: trabecular-ciliary process distance; ICPD: iris-ciliary process distance

There were no complications during the TCP procedure. After the TCP, anterior chamber cells were present in all the patients, which could be part of the APAC attack itself or it could be due to the TCP. A self-limiting ciliochoroidal detachment was found in 5 patients (39%) by UBM, mostly in the superior and temporal quadrant (Fig. 4). There were no complications during the subsequent cataract surgery or post-cataract surgery. For the control group, there was 1 patient having a posterior lens capsule rupture during phacotrabeculectomy. After surgery, there were 5 patients with a fibrinous membrane in the anterior chamber in the control group, which needed YAG laser to resolve.

## Discussion

In this study, we explored the safety and efficacy of emergency TCP followed by scheduled cataract surgery on treating refractory APAC and described the anterior segment morphological changes of APAC eyes after TCP by UBM, aiming to explore the IOP-lowering mechanism of TCP. The acute attack resolved in all included patients with a major lowering of the IOP. The observed anatomical changes were an increase in ACD, inferior AOD500, and CBTmax, and a decrease in ICPD. Outcomes after 6 months regarding BCVA and IOP control were similar for emergency TCP followed by cataract surgery versus emergency phacotrabeculectomy.

After two reports on TCP in chronic angle closure glaucoma eyes [16, 17], the use of TCP on acute angle closure (AAC) eyes was first reported in 5 AAC cases from two ophthalmic units in the United Kingdom, in which conventional management, including topical and systemic medical treatment, laser iridotomy, and laser iridoplasty, had been unsuccesful [6]. All of their 5 cases achieved a successful control of the IOP and resolution of the acute attack after TCP. TCP was also shown to be safe and effective in reducing IOP in AAC patients secondary to intumescent cataract [5, 7]. The only published paper of TCP on APAC eyes was from our previous study [4], in which the IOP of medically unresponsive APAC eyes decreased but remained over 21 mmHg after TCP in all eyes. In the present study, however, the IOP of all the patients was well controlled. This difference might be the result of different laser settings and/or treatment location (20 × 2000 ms × 2000 mW in the inferior quadrant in the current study versus 20–40 × 1000 ms × 1700–2200 mW in the inferior 180 degrees in our previous study).

In this study, we tried to uncover the mechanism underlying the observed decrease in IOP after TCP in APAC. One possible mechanism explaining the decrease in IOP is shrinkage of the ciliary body. Histologically, TCP can cause coagulation necrosis, tissue contraction, and focal atrophy of ciliary body [18, 19], and clinically shrinkage can be observed during endocyclophotocoagulation treatment. Shrinkage will lead to a backward movement of the lens-iris diaphragm, deepening of the anterior chamber, and re-opening of an appositionally closed angle. Another possible mechanism is a sudden decrease in aqueous humor production, which may also contribute to the resolution of the pupillary block [1]. We found significant changes in four parameters: ACD (increase), AOD500 (increase), ICPD (decrease), and CBTmax (increase). The first three are ‘in between structures’ measures; CBTmax is a ‘structure’ measure. Both shrinkage and a sudden decrease in aqueous humor production will change the ‘in between structures’ measures (ACD, AOD500, and ICPD) in the observed direction. These changes describe the disappearance of the block and support the clinical observation: a huge decrease in IOP and clearance of the cornea. At first sight, the observed increase in CBTmax seems to falsify the shrinkage hypothesis, thus favoring a sudden decrease in aqueous humor production as the primary mechanism. However, we only treated inferiorly and the effect of this treatment was at least as effective as the treatment we performed over a larger area in our earlier study [4], arguing against a sudden decrease in aqueous humor production as the primary mechanism. One possibility is an initial shrinkage resolving the pupillary block followed by swelling due to edema formation. UBM observations performed more frequently than a single observation one day after the treatment, as we did, should be able to further our understanding of the exact mechanism of the beneficial effect of TCP in APAC.

The BCVA of all the APAC eyes improved and there was no vision loss after TCP. The main reason of the improvement might be the disappearance of corneal edema after TCP. TCP was initially introduced for IOP reduction in eyes with little or no visual potential. Nowadays, however, TCP is being used widely in eyes with good vision, although the results vary [16, 17, 20, 21]. Four studies presenting data on visual acuity changes due to TCP [22–25] reported amounts of vision loss after TCP of 2 lines or more in 24% [22], 2 lines or more in 31% [23], unspecified loss in 13% [24], and 3 lines or more in 14% [25]. These values are in line with values reported after trabeculectomy or tube surgery [26]. The absence of vision loss in our series suggests that there was no excessive transient IOP rise directly after TCP (we did not measure IOP directly after the procedure; the first measurement was at day 1 after TCP).

Although there were 2 patients in the control group who needed medications to control IOP, there was no significant difference in IOP and BCVA at 6 months after surgery between the two groups. However, the complications of the control group were more severe than study group. This might be resulted from the cloudy cornea and high IOP during phacotrabeculectomy. The poor visibility would increase the risk of intra-operative complications and the dramatic sudden decrease of IOP during surgery would facilitate the occurrence of post-operative fibrinous membrane. Moreover, even without a difference in final BCVA and IOP, the TCP approach has a major logistic advantage as no emergency surgery has to be performed.

We acknowledge the limitations of our study. First, this was not a randomized clinical trial and the sample size was relatively small. Second, IOP was measured by noncontact tonometry. Although a good inter-device agreement between Goldmann tonometry and noncontact tonometry has been reported, [27, 28] Goldmann tonometry is still the gold standard in IOP assessment. However, Goldmann readings also have their limitations with cloudy, edematous corneas, and given that the treatment-induced IOP changes were very large, the type of tonometer is also less critical. Third, the duration of the IOP-lowering effect of TCP was not evaluated, because TCP was considered a temporary measure to allow for further, final cataract surgery. However, to the best of our knowledge, this is the first study to evaluate the safety and efficacy of TCP followed by cataract surgery on treating APAC and our UBM results can help to better understand the IOP-lowering mechanism of TCP on APAC eyes.

## Conclusions

Our findings suggest that TCP can effectively lower the IOP, deepen the ACD, and widen the inferior angle in APAC. The presumed mechanism is an initial shrinkage of the involved tissues; UBM more frequently performed early after treatment is needed to further our understanding regarding the exact mechanism. However, even without the mechanism being fully uncovered, TCP could be considered a viable option for the initial treatment of APAC and can facilitate the following cataract surgery. Obviously, a further randomized two-armed clinical trial (TCP followed by cataract extraction versus cataract extraction alone) with larger sample size is needed to confirm the promising results in the present study.

## Data Availability

The datasets used and/or analysed during the current study are available from the corresponding author on reasonable request.

## Abbreviations

APAC: Acute primary angle closure
IOP: Intraocular pressure
TCP: Transscleral cyclophotocoagulation
UBM: Ultrasound biomicroscopy
BCVA: Best-corrected visual acuity
ACD: Anterior chamber depth
PD: Pupil diameter
AOD500: Angle-opening distance at 500 μm
IT500: Iris thickness at 500 μm
TCPD: Trabecular-ciliary process distance
ICPD: Iris-ciliary process distance
CBTmax: Maximum ciliary body thickness
IC: Iris curvature
SD: Standard deviation
IQR: Interquartile range
AAC: Acute angle closure
